# Supplementary material for: Design of an Herbal Preparation Composed by a Combination of Ruscus aculeatus L. and Vitis vinifera L. Extracts, Magnolol and Diosmetin to Address Chronic Venous Diseases through an Anti-Inflammatory Effect and AP-1 Modulation
Source: Plants (Basel). 2023 Feb 26;12(5):1051. doi: 10.3390/plants12051051 (PMC10004780; doi:10.3390/plants12051051)
Supplement: Supplementary file 1 [file plants-12-01051-s001.zip › plants-2111122-supplementary.pdf]

Supplementary materials

Design of an herbal preparation composed by a combination of *Ruscus aculeatus* L. and *Vitis vinifera* L. extracts, magnolol and diosmetin to address chronic venous diseases through an anti-inflammatory effect and AP-1 modulation

Figure S1: LC-MS analysis of *Vitis vinifera* seeds and *Ruscus aculeatus* leaf extracts
Figure S2: Western blot analysis of NF-kB in HUVECs nucleus and cytosol after different treatments.

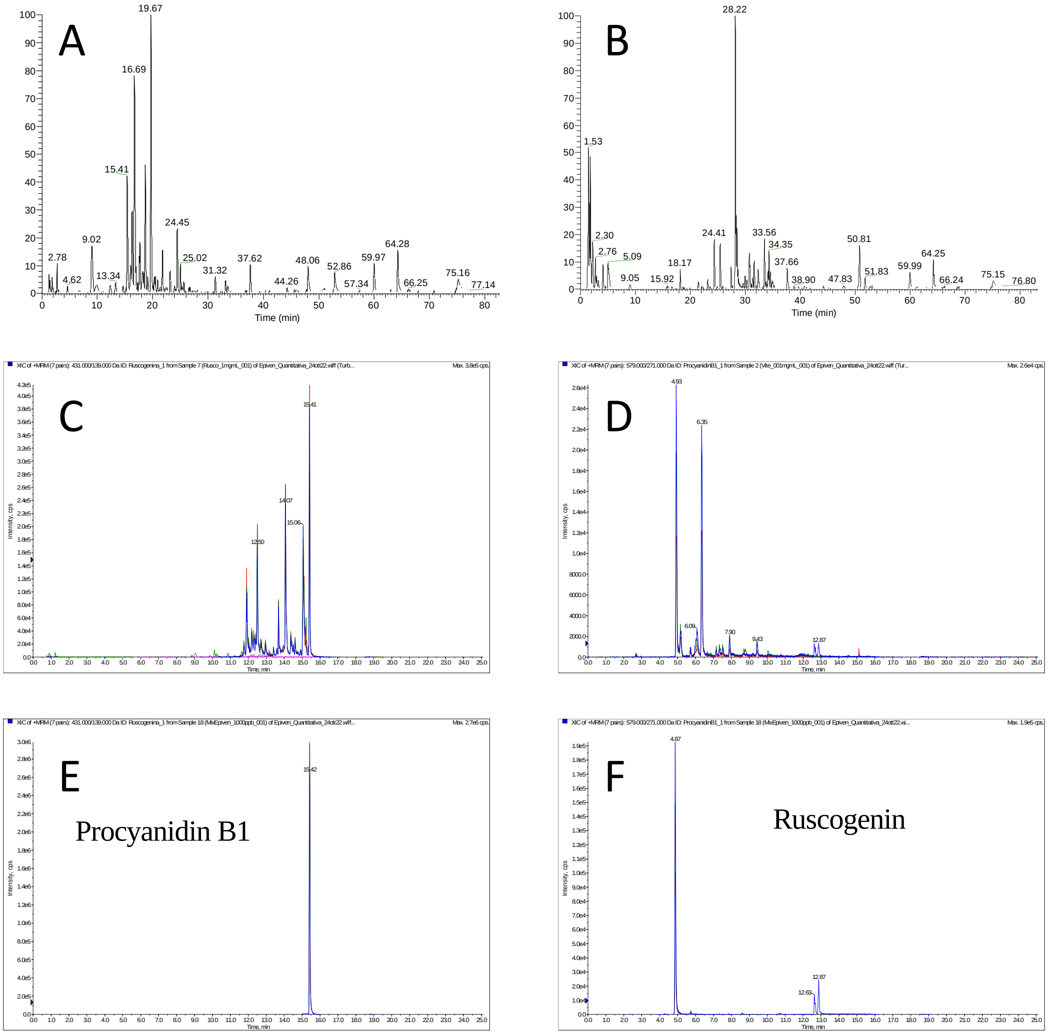

Figure S1: LC-MS analysis of *Vitis vinifera* seeds and *Ruscus aculeatus* leaf extracts. TIC and MRM chromatograms acquired for the extracts of *V. vinifera* (A and C) and *R. aculeatus* (B and D) are reported. MRM chromatograms of pure procyanidin B1 (E) and ruscogenin (F) are also shown.

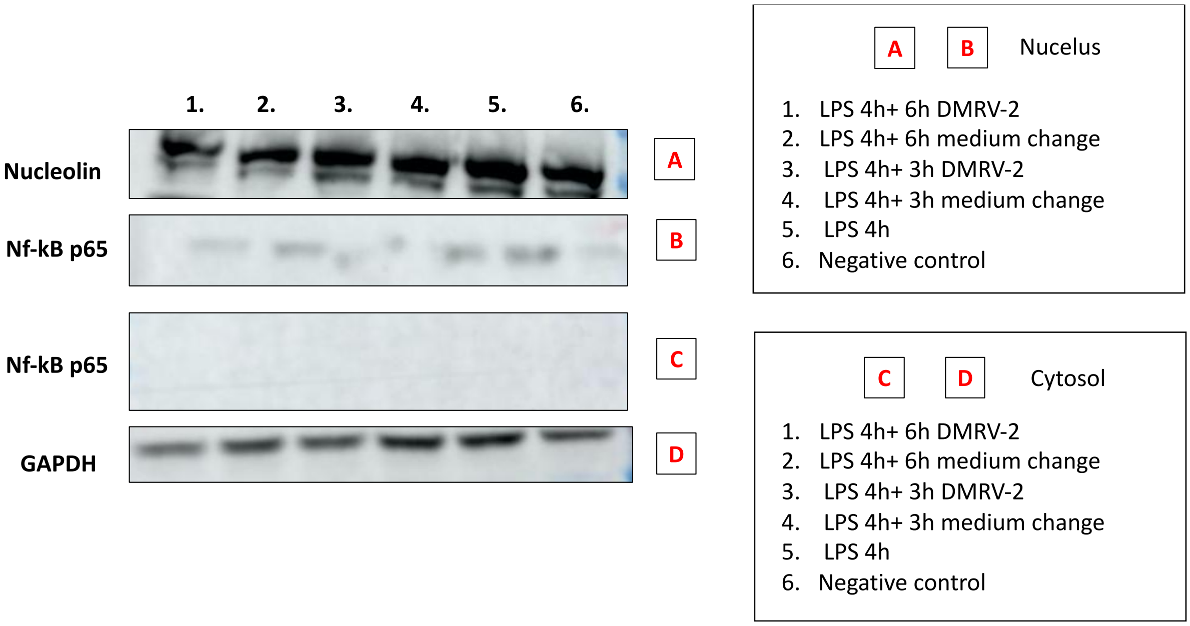

Figure S2: Western blot analysis of NF-kB in HUVECs nucleus and cytosol after different treatments. In particular, the concentration of NF-kB was measured in untreated cells (6), cells subjected to 4h of LPS-induced inflammation (5), and then to incubation with DMRV-2 for 3 h (3) or 6 h (1) or to change of the medium (2 and 4).
